# The Influence of Gender on Isokinetic Knee Performance

**DOI:** 10.7759/cureus.105644

**Published:** 2026-03-22

**Authors:** Meryem Elmajidi, Kenza Ouider, Samira Essoli, M. Mehdi Naciri, Mohammed Chorfi, Asma Elhanafi, Younes Abdelfettah

**Affiliations:** 1 Department of Physical Medicine and Rehabilitation, University Hospital Mohammed VI, Faculty of Medicine and Pharmacy, Cadi Ayyad University, Marrakech, MAR; 2 Department of Clinical Research, University Hospital Mohammed VI, Faculty of Medicine and Pharmacy, Cadi Ayyad University, Marrakech, MAR

**Keywords:** bioelectrical impedance, body composition, gender, h/q ratio, isokinetics, knee

## Abstract

Introduction: Gender-related differences in muscle performance are well documented and are influenced by muscle mass, neuromuscular activity, and hormonal factors. This study aimed to evaluate the influence of gender on isokinetic knee performance and body composition.

Methods: This was a cross-sectional, observational, comparative study involving 30 healthy adults (15 men, 15 women), conducted in Marrakech. Body composition was assessed using multifrequency bioelectrical impedance analysis. Muscle performance was evaluated using an isokinetic dynamometer at various angular velocities and contraction modes (concentric, eccentric).

Results: Men showed higher muscle mass (54.79 ± 2.43 kg vs. 41.14 ± 6.18 kg) and lower fat mass (13.15 ± 3.65 kg vs. 20.61 ± 7.12 kg) than women. Peak torque values were consistently higher in men across all velocities and contraction modes, with statistically significant differences in most testing modalities (all p < 0.001), except for eccentric right extensors at 30°/s (p = 0.065). The hamstring-to-quadriceps (H/Q) ratio did not differ significantly between sexes at 60°/s or 180°/s but was lower in women at 240°/s on the right side (p = 0.028). Muscle mass was strongly correlated with performance (r = 0.522 to 0.877), while fat mass showed weaker negative correlations with peak torque (r = -0.267 to -0.420). ​​​​​​​

Conclusion: Gender has a significant influence on isokinetic performance. The lower H/Q ratio observed in women may have implications for neuromuscular balance and injury prevention. Gender-specific rehabilitation programs are recommended.

## Introduction

Muscle performance is influenced by several factors, with biological sex being a key determinant. It is well known that men usually have higher absolute muscle strength than women, especially in the lower limbs [1]. These differences result from multiple factors, including anatomical features such as muscle mass and tendon insertion; hormonal levels such as testosterone and estrogens; and neuromuscular aspects, including motor recruitment and muscle activation [2,3]. Nevertheless, these differences are not consistent across all types of muscle contractions or evaluation modalities. Some studies also report that men experience greater muscle fatigue [4].

Isokinetic testing is recognized as a reference method for quantifying muscle strength and endurance under controlled dynamic conditions. It provides reproducible measurements of torque at predefined angular velocities [5]. In this context, knee extensor and flexor muscles (quadriceps and hamstrings) are frequently assessed due to their involvement in locomotion, joint stability, and injury prevention, especially in athletic populations [6].

In addition, body composition, particularly lean mass, is a major determinant of muscle strength. Bioelectrical impedance analysis (BIA) is a non-invasive method that estimates this component. Several studies have demonstrated significant correlations between lower limb lean mass and isokinetic performance, with correlation coefficients ranging from 0.6 to 0.9 depending on the measurement technique used (dual-energy X-ray absorptiometry [DXA], D₃-creatine dilution method, Bioelectrical Impedance Analysis [BIA]) [7-9].

Despite these well-established findings, few studies have simultaneously combined isokinetic assessment with bioelectrical impedance analysis to evaluate gender-related differences in muscle performance. To our knowledge, there is limited data available from Morocco and, more broadly, from North Africa on this topic. Understanding these differences may help inform rehabilitation, injury prevention, and training strategies.

Therefore, the present study aimed to investigate the influence of gender on isokinetic knee performance at different angular velocities, in relation to body composition parameters assessed via impedance analysis. A better understanding of the biomechanical and physiological characteristics specific to each sex may help optimize rehabilitation protocols, injury prevention strategies, and individualized athletic programming.

## Materials and methods

Study design and population

This cross-sectional, comparative study was conducted over six months, from September 2024 to February 2025, at the Physical Medicine and Rehabilitation Department of the Mohammed VI University Hospital Center (CHU) in Marrakech. The study included 30 medical residents (15 men and 15 women) who volunteered to participate. Given the exploratory nature of this pilot study, no a priori sample size calculation was performed, and the sample size was determined based on feasibility.

Participants with moderate to high physical activity levels were included. Physical activity was assessed using the French-validated International Physical Activity Questionnaire-Short Form (IPAQ-SF, July 2003) [10], with results expressed in metabolic equivalent of task (MET)-minutes per week. According to IPAQ scoring guidelines, physical activity levels were classified as low, moderate, or high based on MET-minutes/week thresholds.

Elite athletes, defined as individuals competing at a national or international level and training at least five times per week, were excluded. Additional exclusion criteria included a history of trauma, cardiovascular disease, diabetes, severe obesity (body mass index > 30 kg/m²), and use of neurotoxic substances.

Ethical considerations

The study protocol received approval from the local ethics committee of the University Hospital Center of Mohammed VI (no. 154/2024). All participants gave informed consent before participating. Respect for participant dignity, anonymity, confidentiality, and their right to withdraw were upheld throughout.

Data collection

Data collection was performed using a standardized datasheet that included anthropometric measurements such as height, weight, and BMI, along with body composition analysis and isokinetic performance metrics.

Body composition was measured using the Tanita NC980 segmental bioelectrical impedance analyzer (BIA), according to the manufacturer’s guidelines. Participants stood barefoot on electrodes and held handles, with measurements completed within 30 seconds. Participants were instructed to avoid intense physical activity prior to testing; however, hydration status and fasting conditions were not strictly standardized.

Isokinetic testing

Muscle performance was tested with the HUMAC Norm isokinetic dynamometer (CSMi, Stoughton, MA, USA). Participants were seated with their trunks and thighs strapped and their hips at 85°of flexion for stabilization. Knee range of motion was set from 0° to 100° and adjusted to each participant’s anatomy. A standardized 10-minute treadmill warm-up was performed prior to testing, followed by a familiarization pre-test session. The testing protocol included concentric knee flexion-extension contractions at angular velocities of 60°/s (3 repetitions), 240°/s (3 repetitions), and 180°/s (30 repetitions). An eccentric contraction protocol was also performed at 30°/s (3 repetitions) with a force limit of 375 Nm. Testing began with the dominant limb. Standardized rest periods were used between sets. Gravity correction was applied according to the manufacturer’s recommendations.

All assessments were conducted during daytime hours under similar environmental conditions. However, the exact time of day was not strictly standardized across participants.

The fatigue index was automatically calculated by the dynamometer software using the manufacturer’s standard method, which accounts for the decline in peak torque across repetitions. Equipment calibration and verbal encouragement were standardized to minimize measurement bias.

In female participants, the menstrual cycle phase was not controlled, which may have influenced muscle performance. This aspect was considered a limitation of the study.

Statistical analysis

Statistical analysis was conducted with IBM Corp. Released 2019. IBM SPSS Statistics for Windows, Version 25. Armonk, NY: IBM Corp. Descriptive statistics included percentages and frequencies for categorical variables, and means with standard deviations for continuous variables or medians with ranges depending on the distribution’s normality. The normality was tested using the Kolmogorov-Smirnov test; if the p-value exceeded 0.05, the Student’s t-test for independent samples was applied. When the p-value was below 0.05, the non-parametric Mann-Whitney U test was used. For comparing percentages, either the Chi-squared test or Fisher’s exact test was employed as appropriate. Spearman correlation analysis was performed to evaluate relationships between variables. A significance threshold of p < 0.05 was maintained.

## Results

A total of 30 participants were included in the study. The mean age of participants was 24.90 ± 0.75 years. The majority of subjects (96.7%) were right lower-limb dominant, while 3.3% were left dominant. The average body weight was 67.08 ± 9.90 kg. The mean body mass index (BMI) was 23.19 ± 2.89 kg/m².

Baseline characteristics of the study population are summarized in Table 1.

**Table 1:** Baseline characteristics of male and female participants

Variable	Male (n = 15)	Female (n = 15)	Test	p-value	Total (n = 30)
Age (years)	25.13 ± 0.74	24.66 ± 0.72	t = 1.742	0.092	24.90 ± 0.75
BMI (kg/m²)	23.20 ± 2.13	23.19 ± 3.58	t = 0.004	0.997	23.19 ± 2.89
Height (cm)	175.07 ± 5.59	164.80 ± 5.45	U = 23.000	<0.001	169.90 ± 9.90
Weight (kg)	70.84 ± 4.01	63.32 ± 12.51	U = 49.000	0.008	67.08 ± 9.90
Physical activity (categorical)			χ² = 0.144	0.705	
– Moderate	10 (66.7%)	9 (60.0%)			19 (63.3%)
– High	5 (33.3%)	6 (40.0%)			11 (36.7%)
Dominant side			χ² = 1.034	0.309	
– Right	15 (100%)	14 (93.3%)			29 (96.7%)
– Left	0 (0%)	1 (6.7%)			1 (3.3%)

There was no difference between males and females regarding their age (t = 1.742, p = 0.092), BMI (t = 0.004, p = 0.997), or categorical physical activity level (χ² = 0.144, p = 0.705), and the dominant side (χ² = 1.034, p = 0.309). However, statistically significant differences were observed for body weight and height, with men being significantly taller (Mann-Whitney U test = 23.000, p < 0.001) and significantly heavier (Mann-Whitney U test = 49.000, p = 0.008).

In terms of body composition, men showed higher muscle mass than women (54.79 ± 2.43 kg vs. 41.14 ± 6.18 kg). Conversely, women presented greater fat mass compared to men (20.61 ± 7.12 kg vs. 13.15 ± 3.65 kg).

Isokinetic performance assessed at different angular velocities demonstrated consistently higher values in men compared to women across all testing conditions (Mann-Whitney U test).

At 60°/s, men exhibited significantly greater peak torque values for both knee extensors and flexors on the right and left sides (all p < 0.001). No significant sex-related differences were observed for the hamstring-to-quadriceps (H/Q) ratio on either side (right: U = 79.500, p = 0.177; left: U = 98.500, p = 0.574).

At 180°/s, men continued to demonstrate significantly higher peak torque values for both extensors and flexors bilaterally (all p < 0.001). No significant difference was observed for the H/Q ratio at 180°/s on either side (right: U = 76.500, p = 0.140; left: U = 110.000, p = 0.934).

At 240°/s, peak torque values were consistently higher in men for both extensors and flexors on both sides (all p < 0.001). The H/Q ratio was significantly lower in women on the right side (U = 59.000, p = 0.028), while no significant difference was observed on the left side (U = 107.500, p = 0.852).

In eccentric mode at 30°/s, men demonstrated higher peak torque values for flexors bilaterally and for left extensors (right extensors: U = 67.500, p = 0.065; right flexors: U = 24.500, p < 0.001; left extensors: U = 51.000, p = 0.011; left flexors: U = 19.500, p < 0.001). The eccentric H/Q ratio was also higher in men on the right side (U = 61.500, p = 0.036), whereas no significant difference was found on the left side (U = 70.000, p = 0.081).

Fatigue indices at 180°/s did not differ significantly between sexes for either extensors or flexors (all p > 0.05).

Overall, these findings demonstrate that men displayed consistently higher peak torque values than women across all concentric and eccentric conditions, with statistically significant differences observed in most testing modalities, as detailed in Table 2.

**Table 2:** Isokinetic peak torque values of knee flexors and extensors at different angular velocities (concentric 60°/s, 180°/s, 240°/s; eccentric 30°/s; H/Q ratios)

Velocity	Side	Variable	Male	Female	U	p	Total
Concentric/Concentric: 60°/s	right	Peak torque extensors	206.40 ± 31.20	146.53 ± 26.48	15.000	<0.001	176.47 ± 41.66
		Peak torque flexors	97.40 ± 16.42	61.93 ± 15.63	13.500	<0.001	79.67 ± 23.94
		H/Q ratio	47.60 ± 8.08	42.27 ± 8.88	79.500	0.177	44.93 ± 8.77
	left	Peak torque extensors	201.87 ± 37.76	139.60 ± 20.26	14.000	<0.001	170.73 ± 43.46
		Peak torque flexors	92.87 ± 18.76	63.60 ± 13.92	20.000	<0.001	78.23 ± 22.02
		H/Q ratio	46.47 ± 6.37	45.40 ± 6.26	98.500	0.574	45.93 ± 6.23
Concentric/Concentric: 240°/s	right	Peak torque extensors	117.93 ± 40.83	64.87 ± 24.15	17.000	<0.001	91.40 ± 42.60
		Peak torque flexors	55.40 ± 9.72	22.40 ± 10.66	14.000	<0.001	38.90 ± 24.79
		H/Q ratio	46.73 ± 9.72	35.67 ± 11.51	59.000	0.028	41.20 ± 11.88
	left	Peak torque extensors	120.13 ± 36.29	70.33 ± 20.94	14.000	<0.001	95.23 ± 38.58
		Peak torque flexors	51.07 ± 22.99	28.53 ± 10.86	20.500	<0.001	39.80 ± 21.06
		H/Q ratio	41.93 ± 7.35	40.47 ± 10.15	107.500	0.852	41.20 ± 8.74
Eccentric/eccentric: 30°/s	right	Peak torque extensors	259.53 ± 72.61	207.60 ± 58.33	67.500	0.065	233.57 ± 69.89
		Peak torque flexors	120.60 ± 19.48	82.93 ± 25.12	24.500	<0.001	101.77 ± 29.23
		H/Q ratio	48.60 ± 11.06	40.40 ± 8.52	61.500	0.036	44.50 ± 10.56
	left	Peak torque extensors	255.33 ± 42.74	205.53 ± 42.74	51.000	0.011	230.43 ± 56.14
		Peak torque flexors	114.07 ± 18.75	80.20 ± 17.87	19.500	<0.001	97.13 ± 24.91
		H/Q ratio	46.00 ± 8.01	39.80 ± 6.72	70.000	0.081	42.90 ± 7.92
Concentric/Concentric: 180°/s	right	Peak torque extensors	136.73 ± 32.92	90.60 ± 15.49	10.000	<0.001	113.67 ± 34.49
		Peak torque flexors	63.60 ± 13.70	36.60 ± 11.33	8.500	<0.001	50.10 ± 18.47
		H/Q ratio	46.87 ± 5.51	40.27 ± 9.58	76.500	0.140	43.57 ± 8.38
	left	Peak torque extensors	132.40 ± 19.12	88.73 ± 13.59	13.500	<0.001	110.57 ± 27.55
		Peak torque flexors	57.40 ± 7.28	39.33 ± 10.13	14.000	<0.001	48.37 ± 12.63
		H/Q ratio	44.07 ± 7.01	44.33 ± 7.14	110.000	0.934	44.20 ± 6.96
Concentric/Concentric: 180°/s (Fatigue)	right	Fatigue index extensors	35.71 ± 20.23	44.33 ± 10.96	104.500	0.756	37.85 ± 16.47
		Fatigue index flexors	52.31 ± 22.07	40.08 ± 18.62	77.500	0.152	46.19 ± 20.95
	left	Fatigue index extensors	39.29 ± 16.54	37.43 ± 14.05	108.500	0.884	38.36 ± 15.09
		Fatigue index flexors	48.40 ± 16.13	39.29 ± 15.54	69.500	0.078	44.00 ± 16.24

Correlation analysis was performed using Spearman’s rank correlation coefficient (r). Muscle mass demonstrated strong and consistent positive correlations with isokinetic peak torque values across all angular velocities and contraction modes. At 60°/s, correlation coefficients ranged from r = 0.714 to r = 0.795 (all p < 0.001). Similar strong associations were observed at 180°/s (r = 0.754-0.877, all p < 0.001) and 240°/s (r = 0.718-0.801, all p < 0.001). In eccentric mode at 30°/s, muscle mass remained significantly correlated with peak torque values (r = 0.522-0.807, p ≤ 0.003).

Fat mass showed weaker and predominantly negative correlations with peak torque. Correlation coefficients ranged from r = -0.267 to r = -0.420, with some reaching statistical significance (p-values between 0.021 and 0.046), while others remained non-significant.

No significant correlations were observed between body composition parameters and fatigue indices (all p > 0.17). Associations with H/Q ratios were generally weak and inconsistent.

Overall, muscle mass emerged as the variable most strongly associated with isokinetic strength performance. These correlations are detailed in Table 3.

**Table 3:** Correlation between muscle mass, fat mass, and isokinetic performance parameters

			muscle mass		fat mass	
			r	p	r	p
Concentric/Concentric: V60°/s	right	Peak torque extensors	0.795	<0.001	-0.359	0.051
		Peak torque flexors	0.770	<0.001	-0.368	0.046
		H/Q ratio	0.218	0.218	-0.079	0.678
	left	Peak torque extensors	0.749	<0.001	-0.380	0.038
		Peak torque flexors	0.714	<0.001	-0.328	0.077
		H/Q ratio	0.148	0.148	-0.002	0.991
Concentric/Concentric: V240°/s	right	Peak torque extensors	0.801	<0.001	-0.346	0.061
		Peak torque flexors	0.799	<0.001	-0.385	0.035
		H/Q ratio	0.366	0.047	-0.420	0.021
	left	Peak torque extensors	0.718	<0.001	-0.302	0.104
		Peak torque flexors	0.767	<0.001	-0.273	0.145
		H/Q ratio	0.252	0.252	-0.153	0.179
Eccentric/eccentric: V30°/s	right	Peak torque extensors	0.522	0.003	-0.097	0.608
		Peak torque flexors	0.807	<0.001	-0.335	0.070
		H/Q ratio	0.299	0.109	-0.311	0.094
	left	Peak torque extensors	0.630	<0.001	-0.083	0.663
		Peak torque flexors	0.787	<0.001	-0.313	0.092
		H/Q ratio	0.102	0.591	-0.428	0.018
Concentric/Concentric: V180°/s	right	Peak torque extensors	0.784	<0.001	-0.267	0.153
		Peak torque flexors	0.805	<0.001	-0.367	0.046
		H/Q ratio	0.355	0.054	-0.238	0.205
	left	Peak torque extensors	0.754	<0.001	-0.303	0.103
		Peak torque flexors	0.877	<0.001	-0.352	0.056
		H/Q ratio	0.237	0.208	-0.088	0.642
Concentric/Concentric: V180°/s	right	fatigue index extensors	-0.207	0.310	-0.092	0.656
		fatigue index flexors	0.261	0.197	-0.268	0.185
	left	fatigue index extensors	0.019	0.922	0.119	0.545
		fatigue index flexors	0.257	0.178	-0.257	0.178

In summary, men demonstrated consistently higher peak torque values than women across all angular velocities and contraction modes. Significant differences were observed in most testing conditions, while H/Q ratios showed limited differences between sexes, except at higher velocities. Muscle mass was strongly and positively correlated with isokinetic performance, whereas fat mass exhibited weaker and predominantly negative associations. No significant differences were observed in fatigue indices between sexes. Overall, these findings highlight the influence of sex and body composition on isokinetic knee performance.

## Discussion

The present study investigated sex-related differences in knee isokinetic performance and their relationship with body composition in a cohort of healthy young adults, showing that men displayed consistently higher peak torque than women across all concentric and eccentric conditions, while women exhibited lower torque values and a tendency toward reduced hamstring-to-quadriceps ratios at higher angular velocities. These differences occurred despite similar age, BMI, and self-reported physical activity levels, suggesting that body composition, particularly muscle mass, and sex-specific neuromuscular characteristics are major determinants of isokinetic strength in this population.

Correlation analysis further demonstrated that muscle mass assessed by bioelectrical impedance was strongly and positively associated with peak torque of both extensors and flexors across all velocities, whereas fat mass showed weaker negative correlations with performance. Associations with H/Q ratios were generally weak and inconsistent, and no significant associations were observed with fatigue indices.

Our findings that men produce greater isokinetic knee torque than women are consistent with extensive evidence showing that male adults exhibit higher lower-limb strength, largely related to greater muscle cross-sectional area and fiber size. Miller et al. reported that men have larger type II fibers in the vastus lateralis and greater maximal voluntary strength even after normalizing for body size, supporting a structural basis for sex differences in torque production [1]. More recently, Nizam et al. examined isokinetic knee strength across physical activity levels in young adults and showed that men consistently outperformed women in extensor and flexor torque at multiple angular velocities, despite comparable levels of habitual activity [11].

Similarly, Liu et al. provided normative isokinetic knee strength values in a large adult sample and confirmed that sex, along with age, height, and weight, independently predicts extensor torque, with men systematically stronger than women [12]. Lyu et al. recently demonstrated that men show higher lower-limb strength and power than women across multiple functional tests, including isokinetic knee strength, in healthy young adults, reinforcing the robustness of sex-based performance differences [13].

Beyond absolute size, neuromuscular factors also contribute to the sex differences observed in our study. Haizlip et al. reviewed sex-based differences in skeletal muscle kinetics and fiber-type composition, showing that men tend to exhibit faster calcium handling and a higher proportion of fast-twitch fibers, which favor rapid force production during dynamic contractions [3]. Hunter emphasized that anatomical and physiological differences between men and women, including muscle perfusion, motor unit recruitment, and metabolic responses, contribute to distinct neuromuscular performance profiles and fatigability patterns [2]. Clark and Manini introduced the concept of dynapenia to describe the loss of strength not fully explained by loss of muscle mass, highlighting that neural factors and muscle quality also play a critical role in determining maximal torque [4].

These mechanisms likely underlie, at least in part, the superior isokinetic performance of men in our sample, even after accounting for BMI and physical activity level.

In our study, men exhibited greater peak torque at all tested angular velocities, whereas torque decreased for both sexes as movement speed increased, which is in line with the classic force-velocity relationship described by Hill [7] and reproduced in modern isokinetic research. Dvir’s reference text on isokinetics indicates that concentric torque typically declines with increasing angular velocity because the time available for cross-bridge formation is reduced, limiting force production at higher speeds [5].

Our observation that eccentric torque exceeds concentric torque is also well established; Herzog and colleagues showed that muscles can produce 20-60% more force during lengthening contractions due to passive elastic components and cross-bridge dynamics [14]. Gerodimos et al. similarly reported higher eccentric than concentric knee torque in both sexes during isokinetic testing, emphasizing the functional importance of eccentric strength in joint stabilization [15].

The hamstring-to-quadriceps ratio is commonly used as a surrogate of neuromuscular balance around the knee, and in our study, H/Q ratios remained generally below the frequently recommended threshold of 50-60% for concentric testing at 60°/s, with a significantly lower ratio in women at 240°/s on the dominant side. Kellis recently reviewed the clinical utility of H/Q ratios and noted that low ratios are associated with an increased risk of hamstring strain and may compromise knee joint stability, particularly in dynamic sports [16].

In professional football players, Veeck et al. reported that low preseason H/Q ratios were associated with a higher risk of hamstring injury, underlining the clinical relevance of monitoring this parameter even in asymptomatic populations [17]. Furthermore, Shirani Dastjerdi et al. recently showed that simulated match-induced fatigue further reduces H/Q ratios in handball players, suggesting that dynamic neuromuscular imbalances may become more pronounced under fatigue conditions [18].

In our protocol, fatigue indices did not differ significantly between men and women, which is consistent with the notion that sex differences in fatigability are highly task dependent. Hunter’s review indicated that women often display lower fatigability than men during sustained submaximal isometric contractions, but these advantages may be reduced or absent during brief, high-intensity dynamic tasks similar to isokinetic testing conditions [2]. Yoon et al. reported that sex differences in fatigue of dynamic contractions depend on contraction intensity and muscle group, with smaller or inconsistent differences at high workloads [19]. Croix et al. found no major sex effect on fatigue indices during repeated maximal knee extensions when sufficient rest intervals were provided, which may explain the absence of significant sex differences in fatigability in our short isokinetic sets [20].

A central contribution of this study is the combined analysis of body composition and isokinetic performance, showing that muscle mass was strongly and positively correlated with knee peak torque, while fat mass tended to be negatively associated with strength. Cataldi et al. recently examined multiple body composition methods (D₃-creatine dilution [DXA] and BIA) in collegiate athletes and demonstrated strong correlations between leg lean mass and isokinetic knee strength, with correlation coefficients often exceeding 0.6-0.8 [21]. Fukuoka et al. used muscle-localized BIA to track changes in thigh impedance parameters and found that improvements in ML-BIA measures were closely related to gains in performance, supporting the validity of localized bioimpedance indices for monitoring muscle function [22]. Sue et al. showed that BIA-derived skeletal muscle mass significantly predicted leg-press one-repetition maximum in healthy adults, confirming that bioimpedance parameters are meaningful proxies for lower-limb strength [23]. These findings support our observation that BIA-estimated muscle mass is a major determinant of isokinetic torque in young adults.

Several recent studies have linked body composition to isokinetic performance in different populations. Shi et al. compared novice and amateur marathon runners and reported that those with higher lower-limb muscle mass measured by BIA exhibited greater knee extensor strength and better functional performance than runners with lower lean mass [24]. Machado et al. reported that, in futsal players, lower-limb lean mass correlated positively with isokinetic knee torque, while higher fat mass was associated with reduced relative performance, emphasizing the need to optimize body composition for strength and power in sport contexts [25]. Tong et al. examined older adults and found that knee muscle strength was independently associated with both fat and muscle mass, suggesting that excess adiposity and low lean mass jointly contribute to functional limitations [26].

Together, these data parallel our findings by confirming that muscle mass is a strong positive predictor of isokinetic performance, whereas fat mass exerts a detrimental effect on strength across different age groups and activity profiles.

Our observation of weak-to-moderate negative correlations between fat mass and knee torque is in agreement with previous work on the mechanical and metabolic consequences of excess adiposity.

La Roche et al. showed that higher fat mass reduced relatively lower-limb strength and maximal walking performance in overweight older women, primarily due to the added inert load that must be moved during locomotion [27]. Recent work by Shi et al. also indicated that endurance runners with higher body fat percentages displayed lower relative strength and poorer running economy, underlining the performance cost of excess adiposity even in physically active individuals [24].

These findings support the interpretation that fat mass acts as a passive load that impairs relative strength, reinforcing the modest inverse relationship between fat mass and isokinetic torque observed in our sample.

The present study used multifrequency BIA to assess body composition, which is increasingly recognized as a practical, bedside tool for estimating muscle mass and muscle quality.

Da Costa Pereira et al. conducted a recent systematic review and meta-analysis showing that phase angle, a BIA-derived parameter, is a robust marker of muscle quality and functional status across various populations [28]. Hu et al. recently reported that lower phase angle predicts motor function decline in pediatric neuromuscular disease, illustrating that BIA-derived metrics are sensitive to changes in muscle function in both pediatric and adult cohorts [29].

In this context, our findings add to the growing evidence that combining BIA with isokinetic testing can help clinicians interpret strength measures in relation to underlying body composition and muscle quality.

Several aspects strengthen the clinical relevance of our findings. First, our participants were healthy young adults, an age group where early identification of neuromuscular imbalances or suboptimal H/Q ratios may help prevent future injury, especially in those engaging in recreational or competitive sports.

Nizam et al. underscored that even in non-athletes, substantial variability in isokinetic knee strength exists across activity levels, supporting the need for individualized profiling [11]. Second, the strong association between muscle mass and strength in our study reinforces the idea that training interventions aiming to increase lower-limb lean mass may have a direct impact on isokinetic performance and functional capacity, a concept also highlighted in Cataldi and Machado’s work [21,25].

Third, the identification of lower H/Q ratios in women at high angular velocity supports targeted eccentric hamstring strengthening and neuromuscular training for female subjects, in line with recommendations from Kellis and Veeck regarding ACL and hamstring injury prevention [16,17].

This study also has limitations that must be considered when interpreting the results. The sample size was modest and drawn from a single center, which may limit generalizability to other populations with different ethnicities, training, or clinical characteristics. Our cross-sectional design precludes causal inference regarding the effects of changes in muscle or fat mass on isokinetic strength; longitudinal or interventional studies would be needed to confirm these relationships. Body composition was estimated using BIA rather than DXA or magnetic resonance imaging (MRI), which are considered reference methods, although several recent studies have confirmed the good agreement between BIA-derived skeletal muscle mass and more advanced imaging techniques in both healthy and clinical populations.

Finally, our cohort included recreationally active but non-athletic volunteers, all of whom were medical residents, which may introduce a selection bias and limit the generalizability of the findings to the broader population. Different patterns might be observed in elite athletes or in patients with musculoskeletal disorders such as knee osteoarthritis or anterior cruciate ligament (ACL) reconstruction, where pain, joint degeneration, and surgical history may alter strength and body composition relationships.

In summary, this study confirms that men demonstrate superior isokinetic knee strength compared with women, largely driven by greater muscle mass, and shows that muscle mass measured by bioimpedance is strongly and positively correlated with isokinetic performance in healthy young adults. These findings are consistent with recent literature on sex differences in neuromuscular function and the structural determinants of strength, as illustrated by Nizam, Lyu, and Cataldi [11,13,21].

At the same time, our results highlight modest but consistent negative associations between fat mass and strength, supporting previous work showing that excess adiposity impairs relative lower-limb performance. The low H/Q ratios observed in both sexes and the particularly reduced ratio in women at high velocities underscore the need for targeted hamstring strengthening and neuromuscular training to optimize dynamic knee stability and reduce injury risk. Altogether, these findings support an integrated approach combining isokinetic assessment and body composition analysis to guide individualized prevention and rehabilitation strategies in young adults.

## Conclusions

In conclusion, our study highlights the relevance of isokinetic testing, particularly when combined with body composition analysis, in the objective assessment of muscle performance. The observed gender differences, primarily explained by disparities in muscle mass, underscore the need to adapt training and rehabilitation protocols to individual physiological profiles. The consistently low hamstring-to-quadriceps ratios observed in our study support the implementation of targeted hamstring strengthening programs, particularly in the context of anterior cruciate ligament (ACL) injury prevention. Furthermore, the negative impact of fat mass on muscle performance reinforces the value of a comprehensive approach that integrates both muscle strengthening and reduction of adiposity.

These findings align with the principles of personalized medicine, advocating for rehabilitation strategies tailored to each individual's morpho-functional characteristics. From this perspective, a future interventional study conducted on the same cohort could objectively assess the effectiveness of an isokinetics-based targeted rehabilitation program.
